# A Rare Case of a Translocation-Associated Perivascular Epithelioid Cell Neoplasm (PEComa)

**DOI:** 10.1155/2022/7519456

**Published:** 2022-04-21

**Authors:** Kimberly Pereira, Arati A. Inamdar, Aditi Zaveri, Jonathan E. Teitelbaum, Wendy Shertz, Kenneth Belitsis

**Affiliations:** ^1^Department of Pediatrics, RWJBarnabas Health, Monmouth Medical Center, Long Branch, NJ 07740, USA; ^2^Department of Pathology, RWJBarnabas Health, Monmouth Medical Center, Long Branch, NJ 07740, USA; ^3^Monmouth Gastroenterology, Monmouth Medical Center, Long Branch, NJ 07740, USA

## Abstract

A perivascular epithelioid cell tumor (PEComa) is a rare mesenchymal neoplasm composed of perivascular epithelioid cells with distinctive histologic, immunohistochemical, and genetic features. PEComas arising from various anatomical sites have been reported, but gastrointestinal PEComas are extremely rare entities. Here, we discuss the clinical and pathological features of a gastrointestinal PEComa with a transcription factor E3 (TFE3) translocation in a 17-year old adolescent male with a clinical presentation of abdominal pain and gastrointestinal bleeding. Our case report provides insight into this rare entity as well as discusses the pathophysiological aspects of TFE3-SFPQ-associated GI PEComas and their management.

## 1. Introduction 

A gastrointestinal polyp is a tumoral mass that protrudes from a mucous membrane into the lumen of the digestive tract. Hematochezia or the passage of fresh blood per anus, usually in or with stools, is one of the classical features of a juvenile colorectal polyp. A perivascular epithelioid cell tumor (PEComa) is a rare mesenchymal neoplasm composed of perivascular epithelioid cells with distinctive histologic, immunohistochemical, and genetic features [1]. Gastrointestinal PEComas are exceedingly rare entity, and only handful of GI PEComas with TFE3 mutation have been reported [2, 3].

We present a case of a 17-year-old male with hematochezia, abdominal pain, and anemia. The endoscopy revealed a polypoid mass in the sigmoid colon. The histopathological and immunochemical examination confirmed the diagnosis of PEComa. The molecular analysis using next-generation DNA-sequencing technology demonstrated the TFE3-SFPQ rearrangement due to translocation resulting in an in-frame fusion of TFE3 exons 1–4 to SFPQ exons 8–10. Even though TFE3-SFPQ fusion translocation is commonly seen in PEComas, to our knowledge this translocation has never been reported in GI PEComas.

## 2. Case Report

A 17-year-old male presented with abdominal pain and bloody stools for one week. His stools were initially well formed with bright red blood, but over the course of the week, he additionally experienced increased frequency and urgency of defecation along with melena. His abdominal pain was generalized, intermittent, colicky in nature, and not relieved with defection. He denied any fevers, recent illnesses, nausea, vomiting, rash, or joint pain. There was no recent travel, medication use, or sick contacts. There was no history of melena or hematochezia in the past. His past medical history was significant for mild persistent asthma which was well controlled by inhaled corticosteroids. He had a history of dyspepsia a few years ago due to candida esophagitis. His family history was pertinent for rheumatoid arthritis, ulcerative colitis, and celiac disease in his maternal family.

**Figure 1:**
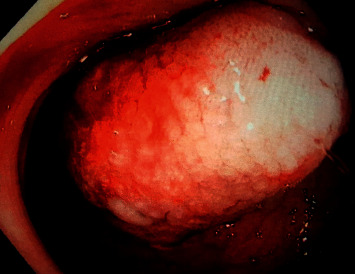
Endoscopic image of the PEComa showing a submucosal polypoid mass with surface ulceration located in the sigmoid colon.

A day prior to presentation, a complete blood count (CBC) revealed a hemoglobin level of 14 mg/dl, computerized tomography (CT) abdomen with oral contrast was normal, and stool cultures were negative. A repeat CBC revealed a hemoglobin level of 12.2 mg/dl with no leukocytosis but with slightly elevated C-reactive protein (CRP) of 25 mg/L (normal < 7 mg/L). Intravenous H_2_ receptor antagonist resulted in improvement in his abdominal pain, but repeat CBC revealed a further decrease in hemoglobin to 10.7 mg/dl the following day. Meckel's scan was negative. The tumor markers were not evaluated. An esophagogastroduodenoscopy was performed and showed no evidence or source of bleeding. A colonoscopy revealed a single large bleeding, pedunculated sigmoid polyp measuring 2 cm × 1.5 cm × 1.5 cm (Figure 1). The mass was endoscopically removed and sent for histopathological evaluation. The remainder of the colon and terminal ileum were normal. Furthermore, lymph nodes were assessed and found to be benign and hence were not submitted for the pathological assessment.

**Figure 2:**
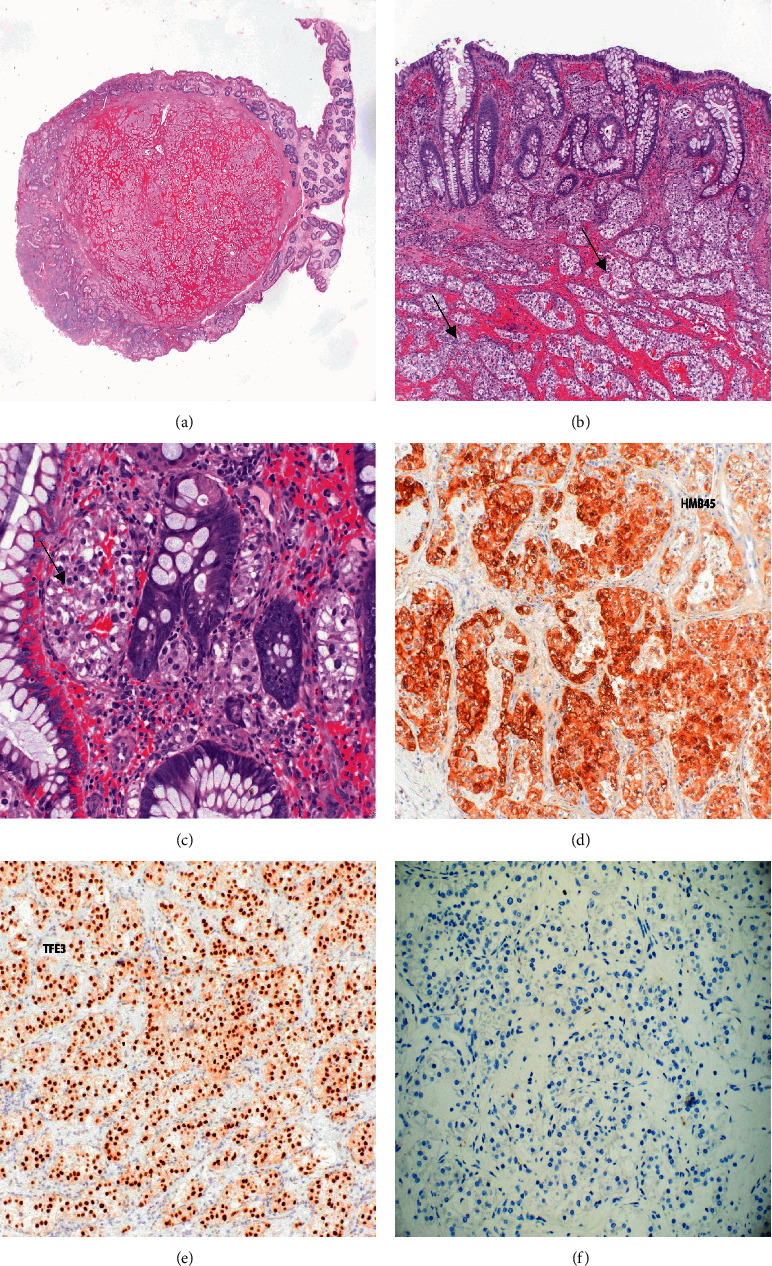
(a) TFE3 rearranged PECOma: low power. (b, c) High-power image of the polyp showing nested lesional cells (arrows) with clear cytoplasm and round nuclei in mucosa and submucosa of colon. (d, e) Lesional cells showed positive immunoreactivity for HMB-45 and TFE3, while (f) negative reactivity for Melan-A.

The pathological and microscopic examination of the resected specimen from the sigmoid colon revealed a polypoid mass with a stalk measuring 2.0 × 1.5 × 1.3 cm (Figure 2(a)). Further evaluation showed lesional cells composed of nested pattern with clear to eosinophilic granular cytoplasm, well-defined cell borders, and round nuclei with prominent nucleoli involving the colonic mucosa and submucosa (Figures 2(b) and 2(c)). The resection margins were free of any lesional cells and measured 0.5 cm from the lesional cells. The immunohistochemical stains showed positive reactivity for HMB-45 (Figure 2(d)) and TFE3 (Figure 2(e)), with negative reactivity for pan-cytokeratin (AE1/AE3), PAX8, synaptophysin, chromogranin, SMA, desmin, CD117, CD68, CD34, and Melan-A (Figure 2(f)). Based on the morphological features, the differentiation diagnosis included alveolar soft part sarcoma and PEComa. PAS with diastase failed to show characteristic crystals of alveolar soft part sarcoma. No mitosis or necrosis was identified. Considering the tumor morphology and staining pattern, the diagnosis of TEF3 rearranged PEComa was rendered. The next-generation DNA-sequencing technology based mutational analysis using MSK-IMPACT assay was performed on the formalin-fixed tumor cells. This assay detects single-nucleotide variants and small insertions and deletions (<30 bp) in protein coding exons of 505 gene panel. The assay demonstrated the TFE3-SFPQ rearrangement due to translocation resulting from an in-frame fusion of TFE3 exons 1–4 to SFPQ exons 8–10. No other significant mutation or copy number alterations were detected. The microsatellite instability MSIsensor score was 0.1, indicating MSI stable status. Random biopsies taken from the upper endoscopy and colonoscopy were normal. Abdominal CT scan revealed no evidence of metastasis.

## 3. Discussion

The term “perivascular epithelioid cells” was first coined by Zamboni et al. [4]. Perivascular epithelial cells are a unique entity having no known “normal” tissue variant discovered to date. It has been described as early as 1992, where it was noted to be consistently present in a variety of tumors including clear cell sugar tumors of the lung and angiomyolipomas. In 2002, the World Health Organization defined PEComas as “mesenchymal tumors composed of histologically and immunohistochemically distinctive perivascular epithelioid cells.” Thus, the umbrella term “PEComa” includes angiomyolipomas (AML), clear-cell “sugar” tumors (CCST), lymphangioleiomyomatosis (LAM), and clear-cell myomelanocytic tumor of uterine ligaments, as well as less well-described clear cell tumors of other anatomic locations such as uterus, colon, and soft tissues. The term perivascular epithelioid cell tumors, not otherwise specified (PEComas-NOS), has been coined to described this latter phenotype. Within this category, GI PEComas represent up to 25% of the burden [5].

Histologically, a perivascular epithelioid cell is made up of a large nucleus and abundant cytoplasm. A distinctive feature for all PEComas is their immunoreactivity for both melanocytic (HMB-45 and⁄or Melan-A) and smooth muscle (actin and⁄or desmin) markers [6]. Our patient's PEComa was positive for HMB-45 antibodies but was negative for any smooth muscle markers. The anti-HMB-45 antibody reacts against an intracytoplasmic antigen present in immature melanosomes [7]. Folpe et al. [8] suggested criteria to classify PEComas into benign, of uncertain malignant potential, and malignant. They proposed that a malignancy was predicted by the presence of 2 or more of the followings: tumor size greater than 5 cm, infiltrative tumor border, high nuclear grade, and cellularity; more than 1 mitosis/50 high-power fields, tumor necrosis, and vascular invasion. The differential diagnosis of these rare tumors includes clear cell sarcoma of soft parts, metastases from malignant melanoma, primary or metastatic epithelioid gastrointestinal stromal tumor, alveolar soft part sarcoma, leiomyosarcoma with HMB-45 expression, and paraganglioma [9].

PEComas have been described in the pediatric population in a variety of locations, namely, the heart, soft tissue, kidneys, urinary bladder, small and large intestine, rectum, skin, ligamentum teres, uterus, vagina, and bone. In the gastrointestinal tract, they are found in the colon, small intestine, rectum, and rarely in the stomach. Most GI PEComas visible on CTs are a well-demarcated homogenous mass. A PubMed search using the key-words “Pediatric” and “PEComa” resulted in 17 cases in children less than 18 years of age, with 5 of these cases occurring in the gastrointestinal tract. Two of the cases involved the ascending colon, one in a 7-year-old boy, whose PEComa was positive for HMB-45 [10] and the other in a 5-year-old boy, whose PEComa tested positive for HMB-45, desmin, and vimentin [11]. One case involved the tongue of a 7-year-old girl, whose tumor was also positive for HMB-45 and SMA [12]. The other two cases involved the transverse colon of an 8-year-old girl, whose tumor was positive for HMB-45, Melan-A, MiTF, c-KIT, and NSE [13] and the rectum of a 15-year-old girl, whose tumor tested positive for HMB-45 and diffusively positive for progesterone, NSE, and cyclin [9]. A review of over 50 case reports describing GI PEComas specifically shows a tendency to affect patients in the mid-thirties with a female predilection and the colon being the most common site. Interestingly, our case showed negative staining for Melan-A but positive staining for HMB-45 (Figures 2(f) and 2(d), respectively). Sugar tumor, clear cell (CSST), lymphangioleiomyomatosis (LAM), and angiomyolipoma (AML) PEComas are also strongly present in concurrence with tuberous sclerosis (TSC), an autosomal-dominant condition involving the TSC gene on chromosome 9. Within the PEComa-NOS category, uterine PEComas have also been described to show genetic concordance with tuberous sclerosis. However, GI PEComas remain unique in that there is no reported case of a GI PEComa in the context of tuberous sclerosis. No clinical history of tuberous sclerosis was evident in our patient.

The molecular analysis of PEComas from various anatomical sites has shown to possess TFE3 gene fusion [14]. TFE3 is a member of the MiTF family of transcription factors and such TFE3 translocation-associated PEComas often demonstrate alveolar architecture and epithelioid cytomorphology. In addition, they stain strongly for TFE3 and HMB-45 while expressing minimal muscle markers [2]. A single case report of TFE3-SFPQ-associated bladder PEComa with benign course has been reported previously [15]. PEComa of the GI tract appears to show variable biological behavior, ranging from benign tumors to aggressive high-grade sarcomas with metastasis irrespective of TFE3 rearrangement. In one case report, a 31-year-old female with malignant gastrointestinal PEComa metastasizing to liver was shown to harbor TFE3-SFPQ rearrangement. Usually, TSC and non-TSC associated high-grade PEComas are treated with a combination of mTOR inhibitors [16] and exemestane, but in this case report, tumor was responsive to only anti-VEGFR TKI Apatinib therapy [17]. Overall, more studies on the genetic precursor and molecular activity of GI and non-GI PEComas are necessary to understand the clinical course and biological behavior. The tumor cells in our patient demonstrated an in-frame fusion of TFE3 exons 1–4 to SFPQ exons 8–10, which has never been reported in a GI PEComas.

Clinically, PEComa is often a diagnostic challenge due to the nonspecificity of symptoms. As seen in our case, the most common presenting symptoms of GI PEComas are abdominal pain and/or rectal bleeding. Our patient's tumor was benign with no lymph node involvement. He had an abdominal CT done which was normal. A follow-up phone call made a year after initial presentation revealed that the patient was doing well and had no abdominal symptoms. He was counselled on following up with a gastroenterologist to assess need for further surveillance.

It is important to have a GI PEComa on the differential diagnosis of a solitary colonic polyp. Histologically, given its epithelioid appearance, a GIST is its most common imitator. However, immunohistochemical melanocytic markers (HMB-45 and Melan-A) are usually positive in PEComas and always negative in GIST.

Limited data are available on the optimal management and follow up of PEComas, but surgical resection is the mainstay of treatment. Majority of PEComa cases are benign. Only few reported PEComa cases had advanced disease course and showed poor prognosis [3, 7, 8]. Chemotherapy has been tried by Rigby et al. [18] in an 11-year-old girl with metastatic renal PEComa without much success. She had presented with two large abdominal masses in the left flank and epigastrium and left supraclavicular lymphadenopathy. The tumor did not respond to an initial treatment of chemotherapy, including dacarbazine, BiCNU, and vincristine. A trial of imatinib mesylate based on the expression of c-KIT was also unsuccessful. While surveillance of GI PEComas are warranted, especially in those with malignant histology, the interval and treatment modality vary from case to case. The recent study underscores the utility of mTOR inhibitors and anti-VEGR therapy for high-grade/advanced PEComas [15]. This case serves as an example of a rare case of TFE3-SFPQ fusion associated GI PEComa that has never been reported.

## References

[1] Fletcher C. D. M., Bridge J. A., Hogendoorn P., Martens F. (2013). *World Health Organization Classification Tumors of Soft Tissue and Bone*.

[2] Lu B., Wang C., Zhang J. (2015). Perivascular epithelioid cell tumor of gastrointestinal tract. *Medicine (Baltimore)*.

[3] Shi H.-Y., Wei L.-X., Lu Sun L., Guo A.-T. (2010). Clinicopathologic analysis of 4 perivascular epithelioid cell tumors (PEComas) of the gastrointestinal tract. *International Journal of Surgical Pathology*.

[4] Zamboni G., Pea M., Martignoni G. (1996). Clear cell “sugar” tumor of the pancreas. *The American Journal of Surgical Pathology*.

[5] Armah H. B., Parwani A. V. (2009). Perivascular epithelioid cell tumor. *Archives of Pathology & Laboratory Medicine*.

[6] Hornick J. L., Fletcher C. D. M. (2006). PEComa: what do we know so far?. *Histopathology*.

[7] Pisharody U., Craver R. D., Brown R. F., Gardner R., Schmidt-Sommerfeld E. (2008). Metastatic perivascular epithelioid cell tumor of the colon in a child. *Journal of Pediatric Gastroenterology & Nutrition*.

[8] Folpe A. L., Mentzel T., Lehr H.-A., Fisher C., Balzer B. L., Weiss S. W. (2005). Perivascular epithelioid cell neoplasms of soft tissue and gynecologic origin. *The American Journal of Surgical Pathology*.

[9] Ryan P., Nguyen V.-H., Gholoum S. (2009). Polypoid PEComa in the rectum of a 15-year-old girl. *The American Journal of Surgical Pathology*.

[10] Park S. J., Han D. K., Baek H. J. (2010). Perivascular epithelioid cell tumor (PEComa) of the ascending colon: the implication of IFN-*α*2b treatment. *Korean Journal of Pediatrics*.

[11] Gross E., Vernea F., Weintraub M., Koplewitz B. Z. (2010). Perivascular epithelioid cell tumor of the ascending colon mesentery in a child: case report and review of the literature. *Journal of Pediatric Surgery*.

[12] Bajpai M. (2018). Non-sporadic PEComa simulating myolipoma of tongue, in a pediatric patient with immunohistochemical analysis using HMB-45 and SMA. *Journal of the College of Physicians and Surgeons Pakistan*.

[13] Pizzi M., Di Lorenzo I., D’Amore E. S., D’Angelo P., Alaggio R. (2014). Pediatric gastrointestinal PEComas: a diagnostic challenge. *Pediatric and Developmental Pathology*.

[14] Doyle L. A., Hornick J. L., Fletcher C. D. M. (2013). PEComa of the gastrointestinal tract. *The American Journal of Surgical Pathology*.

[15] Chen X. F., Yeong J., Chang K. T. E. (2018). TFE3-Expressing epithelioid rich perivascular epithelioid cell neoplasm (PEComa) of the bladder with unusual benign course. *Annals of Clinical and Laboratory Science*.

[16] Sanfilippo R., Jones R. L., Blay J.-Y. (2019). Role of chemotherapy, VEGFR inhibitors, and mTOR inhibitors in advanced perivascular epithelioid cell tumors (PEComas). *Clinical Cancer Research*.

[17] Xu J., Gong X.-L., Wu H., Zhao L. (2020). Case report: gastrointestinal PEComa with TFE3 rearrangement treated with anti-VEGFR TKI Apatinib. *Frontiers in Oncology*.

[18] Rigby H., Yu W., Schmidt M. H., Fernandez C. V. (2005). Lack of response of a metastatic renal perivascular epithelial cell tumor (PEComa) to successive courses of DTIC based-therapy and imatinib mesylate. *Pediatric Blood & Cancer*.

